# Effect of Balance Taping Using Kinesiology Tape and Cross Taping on Shoulder Impingement Syndrome: A Case Report

**DOI:** 10.3390/medicina55100648

**Published:** 2019-09-26

**Authors:** Jung-Hoon Lee, Im-Rak Choi

**Affiliations:** 1 Department of Physical Therapy, College of Nursing, Healthcare Sciences and Human Ecology, Dong-Eui University, Busan 47340, Korea; 2 Department of Rehabilitation Therapy Team, Sports Exercise Therapy Center, Good Samsun Hospital, Busan 47007, Korea; irchoi@hanmail.net

**Keywords:** shoulder impingement syndrome, balance taping, kinesiology tape, cross tape, shoulder pain

## Abstract

*Background and objectives:* Shoulder impingement syndrome (SIS) is the most common disorder among people with shoulder pain. The purpose of this case report was to investigate the effect of the combined application of balance taping using kinesiology tape and cross taping on a part-time worker with SIS. *Case Report:* Combined balance taping and cross taping was applied for 3 weeks (average, 16 hours per day) on a part-time worker with severe pain and a limited range of motion (ROM) in the shoulder who had visual analogue scale (VAS) pain scores of 7 and 8 out of 10 for shoulder flexion and abduction, respectively, and pain and disability scores of the Shoulder Pain Disability Index (SPADI) of 37 out of 50 and 29 out of 80, respectively. After the combined application of balance taping and cross taping, the VAS pain scores for shoulder flexion and abduction decreased from 7 to 0 and from 8 to 0, respectively, and the ROM increased to a normal range. The SPADI pain score decreased from 37 to 2, and the disability score decreased from 29 to 1. Shoulder activity level also increased, and the patient was able to return to his part-time job. *Conclusions:* We suggest combined application of balance taping and cross taping as an effective treatment for part-time workers with SIS.

## 1. Introduction

Shoulder impingement syndrome (SIS) is the most common disorder among people with shoulder pain, with a prevalence of 36% among all shoulder disorders [1], and usually occurs in people who engage in physically demanding work or perform highly repetitive shoulder movements [2]. SIS is caused by structural factors (such as anatomical abnormalities of the coracoacromial arch and humerus head), shoulder joint instability, and functional factors (such as muscle function changes around the rotator cuff or scapula and muscle imbalance) [3,4]. SIS is caused by humeral head translation [5] due to weakness in the serratus anterior and middle and lower trapezius muscles [6] or instability of the glenohumeral joint. Loss of posterior tilt, external rotation, and upward rotation of the scapula decreases subacromial volume, causing compression of the rotator cuff tendon [7]. SIS is associated with intensive shoulder work [8], and shoulder elevation by forceful exertions for a long period at work is a risk factor [9].

Balance taping, which is based on the anatomical structure and approach of muscles, joints, and nerves, involves application of kinesiology tape over the structure that causes the symptoms in order to balance the human body [10]. Previous investigations have found various benefits of using balance taping. Specifically, balance taping using kinesiology tape in amateur university football players with hamstring injuries and traumatic knee pain decreased the knee pain [11]. Additionally, balance taping for acute nonspecific lower back pain also decreased the pain and increased the range of motion (ROM) [12]. Cross taping is the application of cross tape to acupuncture points or high-muscle-tone points to treat various musculoskeletal disorders [13]. In a previous study, cross taping before menstruation alleviated menstrual pain [14]. Another investigation found that cross taping to the trigger point of the upper trapezius muscle relieved pain [15]. However, there is no research on the application of balance taping and cross taping to musculoskeletal disorders such as SIS.

The purpose of this case report was to investigate the effect of the combined application of balance taping using kinesiology tape and cross taping on a part-time worker with SIS who lifts >5 kg objects.

## 2. Case Report

A 27-year-old part-time worker experienced shoulder pain, which started approximately 2 months prior to presentation, during shoulder abduction or when elevating objects with his right upper arm (the dominant side), as he performed his part-time job which required lifting and moving >5 kg objects. He was diagnosed with SIS at a local clinic, but he continued with his part-time job and related activities of daily life without any shoulder treatment. Written informed consent was obtained from the patient for this study.

In the initial assessment, the visual analogue scale (VAS) pain scores were 7 and 8 (0 = no pain; 10 = worst pain) for right shoulder flexion and abduction without resting, respectively. There was no swelling around the right shoulder. The ROM of the shoulder was measured using a goniometer by an assessor with more than 10 years’ experience in measuring ROM with a goniometer: flexion, 134° (normal, 180°); extension, 50° (normal, 60°); abduction, 120° (normal, 180°); external rotation, 80° (normal, 90°); internal rotation, 24° (normal, 90°) [16]. The grades in the manual muscle testing (MMT) of the shoulder were fair for flexion, good for extension, fair for abduction, good for external rotation, and fair for internal rotation (normal = full ROM possible against gravity and maximal resistance; zero = no contraction at all) [17]. 

In the Patient-Specific Functional Scale (PSFS), which is a useful tool for assessing functional status [18], the score of two items was 8 out of 20 (0 = unable to perform the activity; 10 = able to perform the activity) (Table 1). In the Shoulder Pain Disability Index (SPADI), the pain score of 5 items was 37 out of 50 (0 = no pain; 10 = worst pain imaginable) and the disability score of 8 items was 29 out of 80 (0 = no difficulty; 10 = very difficult that help is required). The SPADI, which is a self-reporting questionnaire for measuring shoulder pain and function, was developed for simple assessment of quality of life, pain, and disability associated with shoulder disorder [19], and its reliability and validity have been proven already [20].

Prior to balance taping and cross taping for SIS, contact and movement tests proposed by Lee and Choi [10] were performed. Contact and movement tests, which are used to determine if pain is reduced or increased with ROM, are performed by touching the balance taping or cross taping target area with the palm or finger before applying balance taping or cross taping [10]. First, the tester manually externally rotated the patient’s right arm (contact test) and then elevated the patient’s arm (movement test) [10]. Second, the palm of the tester was placed on the skin over the serratus anterior muscle (contact test) and the tester elevated the patient’s arm (movement test), again confirming the reduced shoulder pain and increased ROM. Third, a finger of the tester was placed on a point on the levator scapulae muscle [21] (contact test) and the tester elevated the patient’s arm (movement test). The results of the contact and movement tests confirmed the reduced shoulder pain using the patient’s VAS pain scores and increased ROM on examination with the goniometer by the same assessor.

**Figure 1:**
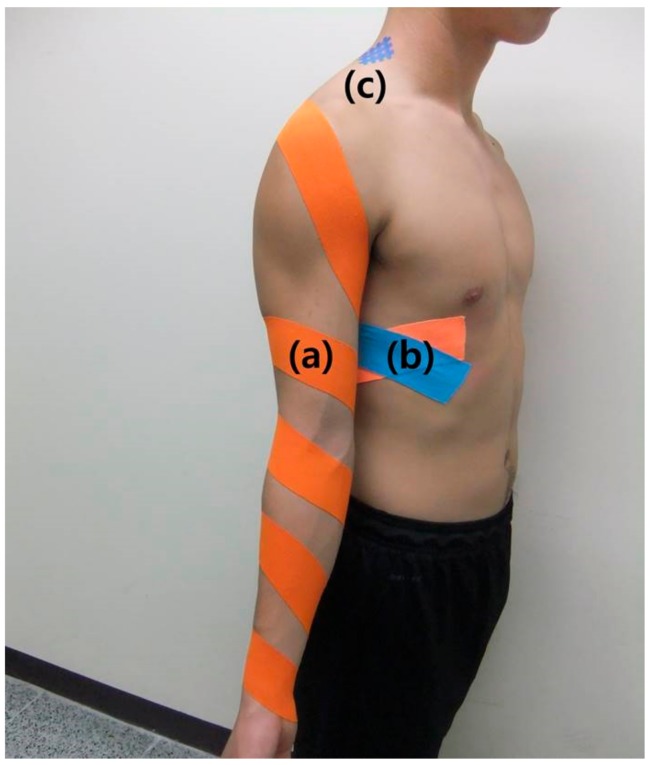
Balance taping using kinesiology tape and cross taping for shoulder impingement syndrome for 1 week. (a) external rotation taping, (b) serratus anterior taping, (c) levator scapulae cross taping.

Based on the results of the contact and movement tests, balance taping and cross taping for SIS were applied immediately as follows. First, after manually externally rotating the patient’s right arm, kinesiology tape (BB Tape; WETAPE, Pyeongtaek, Korea) was applied from the medial part of the wrist to the scapula spine. The tape was stretched by approximately 10% and applied diagonally from the distal to proximal part while applying external rotation (Figure 1a). Second, kinesiology tape was applied to the serratus anterior muscle, where the tester’s palm was placed, to form an X shape, with the tape stretched by approximately 20% (Figure 1b). Third, the cross tape was applied to the point on the levator scapulae muscle where the tester’s finger was placed with four lines of cross tape slanted to the left at a 45° angle [22] (Figure 1c).

One week later, contact and movement tests [10,11] were performed again before reapplying the balance taping and cross taping for SIS to verify the additional balance taping target areas. First, the tester contacted the skin over the supraspinatus muscle with the palm (contact test) and elevated the patient’s arm (movement test) [10]. Second, the tester contacted the skin over the anterior and posterior fibers of the deltoid with the palm (contact test) and elevated the patient’s arm (movement test) [10]. Third, the tester manually retracted the patient’s shoulders (contact test) and elevated the patient’s arm (movement test) [10]. Fourth, the right arm was externally rotated, and contact and movement tests were performed on the serratus anterior and levator scapulae muscles [10]. The results of the contact and movement tests were identical to and confirmed the reduced shoulder pain and increased ROM in the contact and movement tests conducted 1 week before.

**Figure 2:**
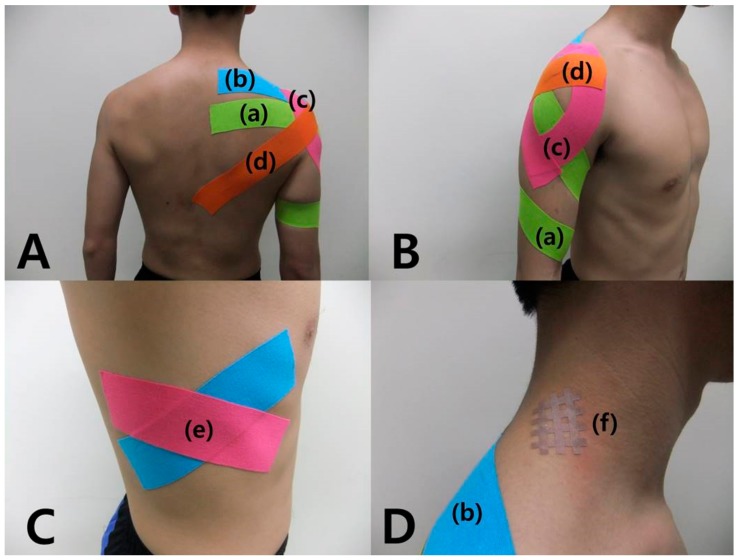
Balance taping using kinesiology tape and cross taping for shoulder impingement syndrome for 2 weeks. (**A**) posterior view; (**B**) lateral view; (**C**) serratus anterior muscle; (**D**) levator scapulae muscle crosstaping. (a) external rotation taping, (b) supraspinatus taping, (c) deltoid taping, (d) retraction taping, (e) serratus anterior taping, (f) levator scapulae cross taping.

Based on the results of the additional contact and movement tests, additional balance taping and cross taping for SIS was applied immediately. First, to maintain external rotation of the right arm, kinesiology tape was applied from the medial part of the elbow to the scapula spine while manually forcing external rotation of the patient’s arm. The tape was stretched by approximately 10% and applied diagonally from the distal to proximal part while applying external rotation (Figure 2a). Second, kinesiology tape was applied to the supraspinatus muscle (from the acromion to the scapula medial border) where the tester’s palm made contact, with the tape stretched by approximately 20% (Figure 2b). Third, kinesiology tape was applied to the anterior and posterior fibers of the deltoid where the tester’s palm made contact, with the tape stretched by approximately 20% [10] (Figure 2c). Fourth, kinesiology tape was applied, with the shoulders retracted, from the anterior aspect of the acromion to the spinous process of the 10th thoracic vertebra, with the tape stretched by approximately 30–40% (Figure 2d). Fifth, kinesiology tape was applied to the serratus anterior muscle, where the tester’s palm made contact, to form an X shape, with the tape stretched by approximately 20% (Figure 2e). Sixth, the cross tape was applied to the point on the levator scapulae muscle where the tester’s finger was placed with four lines of cross tape slanted to the left at a 45° angle [22] (Figure 2f).

The beginning and end of the kinesiology tape (approximately 2–3 cm) were applied unstretched to prevent skin irritation [10,11,23,24]. Kinesiology tape and cross tape were applied for approximately 16 hours daily and replaced with new ones the following day for 3 weeks to achieve the progressive effects of taping as observed in a previous study [11], with subjects visiting the laboratory each day for reapplication of the tape.

After repeated application of balance taping and cross taping for a total of 3 weeks, including the initial week, without other treatment or medication such as analgesics or anti-inflammatory drugs, the VAS scores decreased from 7 to 0 and from 8 to 0 for shoulder flexion and abduction, respectively. Recovery of a normal shoulder ROM was achieved, with an improvement from 134° to 180° for flexion, 50° to 60° for extension, 120° to 180° for abduction, 80° to 90° for external rotation, and 24° to 90° for internal rotation. Shoulder MMT grades also recovered to normal for flexion, extension, abduction, external rotation, and internal rotation.

The PSFS score increased from 8 to 19 out of 20 (Table 1). The SPADI pain score decreased from 37 to 2 out of 50, while the SPADI disability score also decreased from 29 to 1 out of 80. The patient was able to return to his part-time job without shoulder pain or dysfunction.

## 3. Discussion

In this case, the combined application of balance taping using kinesiology tape and cross taping for 3 weeks on a patient with SIS eliminated pain, improved the ROM and muscle strength, and allowed the patient to resume his part-time job.

The application of kinesiology tape enhances proprioception by stimulating cutaneous mechanoreceptors [25,26]. In a recent study, kinesiology tape was applied to the quadriceps muscles of healthy, asymptomatic participants [27]. Moreover, when kinesiology tape was applied to the quadriceps muscles of athletes with muscle fatigue, tactile stimulations and the elasticity of kinesiology tape increased muscle strength by stimulating the stretch and recoil properties of the skeletal muscles [28]. 

SIS has been reported to occur due to structural problems, such as acromion deformity, bony growth, or soft tissue inflammation, and functional problems, such as superior translation of the humeral head and muscle imbalance [29,30]. When the arm is elevated, the muscle activity in the upper trapezius increases and scapular external rotation decreases, which cause changes in muscle activities that could exacerbate SIS [31]. The serratus anterior is an important muscle that originates from the scapular medial border and inserts into the rib cage [32]. It plays an essential role for scapular movement as well as scapular stabilization [33]. It is also involved in scapular protraction and upward rotation, as well as scapular posterior tilt at the end range [32]. Most people with a shoulder injury tend to show low muscle activation in the serratus anterior [34]. In this case report, the application of kinesiology tape to the serratus anterior may have affected muscle activation, whereby an improvement in shoulder pain and ROM may have been achieved by increased scapular upward rotation and posterior tilt.

Generally, scapular internal rotation occurs in the early stage of shoulder flexion on the sagittal plane [35], while external humeral rotation appears at the end range of shoulder flexion [36]. Because external humeral rotation occurs together with glenohumeral joint abduction, the lateral greater tubercle can pass behind the acromion, preventing impingement of the bursa in the subacromial space or the rotator cuff [32]. However, when external humeral rotation is insufficient, the scapulohumeral rhythm could change while the scapular posterior tilt could be reduced [37]. Lopes et al. [31] reported that scapular dyskinesis and subacromial impingement can cause a decline in shoulder function by reducing scapular external rotation. In the present study, external rotation taping of the upper limb may have prevented the impingement of the bursa in the subacromial space and the rotator cuff by improving humeral external rotation while also improving shoulder pain and ROM through scapulohumeral rhythm and scapular posterior tilt improvement.

A rounded shoulder posture exhibits a deformation in the kinematics of the scapula, and an imbalance in the shoulder muscles, while also causing subacromial impingement due to the narrowing of the space between the acromion and the supraspinatus and infraspinatus tendons [6]. When kinesiology tape is applied to dysfunctional muscles, tactile stimulations from taping could provide functional improvement by inducing smoother physiologic contraction in the muscles [25]. Moreover, its mechanical effect also improves abnormal alignment. Lee and Yoo [38] reported that applying kinesiology tape to patients with scapular depression syndrome increased scapular height through mechanical correction and reduced upper trapezius tenderness. Kim and Lee [39] also reported that applying kinesiology tape to patients with scapular downward rotation reduced that scapular downward rotation and shoulder pain by its mechanical effect. Han et al. [23] reported that rounded shoulder posture was reduced by the mechanical effect of applying kinesiology tape from the anterior aspects of the acromion to the spinous process of the 10th thoracic vertebra in the scapular retraction position. In the present study, applying kinesiology tape from the anterior aspect of the acromion to the spinous process of the 10th thoracic vertebra in the shoulder retraction position, with the tape stretched by approximately 30–40% for normal shoulder alignment, may have improved shoulder pain and ROM by improving shoulder alignment.

When the arm is elevated in a normal shoulder joint, the humerus moves in the scapular glenoid fossa, generating superior shear force, while the inferior shear force acts to counteract this superior shear force for correct positioning of the humeral head in the scapular glenoid fossa. The deltoid and rotator cuff work together and are referred to as the “force couple” [40]. The supraspinatus, a part of the rotator cuff, plays a role in maintaining the humeral head in the glenoid fossa when the deltoid acts during arm elevation [33]. However, patients with SIS have reduced activities in the deltoid and rotator cuff muscles, whereby SIS appears prominently in the early stage of arm elevation [41]. Kaya et al. [42] reported that applying kinesiology tape to the supraspinatus of patients with SIS improved pain and arm function due to the increased recruitment of motor units to the supraspinatus. Lyman et al. [43] reported that applying kinesiology tape to the deltoid increased the acromiohumeral distance. This may have been due to the occurrence of gliding, instead of translation toward the acromion, from the force couple during glenohumeral abduction after kinesiology tape application to the deltoid [33], which may have increased the acromiohumeral distance. In the present study, applying kinesiology tape may have improved shoulder pain and ROM by increasing the muscle activities of the deltoid and supraspinatus to affect the force couple.

Janda [33] indicated that the weakening of the middle and lower trapezius, serratus anterior, infraspinatus, and deltoid muscles, along with tightness in the upper trapezius, pectorals, and levator scapulae muscles, affects the onset of SIS. Although meta-analyses, systematic reviews, and randomized control studies regarding the therapeutic effect of cross taping on myofascial trigger point are lacking [15], it has been reported that applying cross tape to areas with high tension or tenderness could reduce muscle tension and pain by utilizing electromagnetic flow through the skin [13]. In previous studies, applying cross tape to the trigger point of the upper trapezius muscle alleviated pain [15], whereas applying cross tape to the dominant bicep reduced the delayed onset of muscle soreness and increased functional performance [44]. In the present study, cross taping of the tight levator scapulae point may have reduced shoulder pain and improved ROM by relaxing the tension in the levator scapulae muscle.

This case report has some limitations. First, it is a single case study, and thus, no comparisons with other interventions could be made. Second, the scales used in this study are subjective, and more quantitative measures should be suggested. Third, the activity level of the subject was not fully controlled. Fourth, we did not evaluate the scapulohumeral rhythm at any point in the study. Fifth, we did not determine whether kinesiology tape applied to the serratus anterior affected activation of the serratus anterior, which involves an increase in scapular upward rotation and posterior tilt. Future studies should build upon this case study by addressing these limitations.

Based on the findings in this case report, we suggest that combined application of balance taping using kinesiology tape and cross taping is an effective treatment for SIS and should be explored further.

## Figures and Tables

**Table 1:** Outcomes of the PSFS.

Assessment	Baseline Score	Final Score
PSFS (score)	8/20	19/20
Activity 1: lifting >5 kg object	3/10	9/10
Activity 2: elevating arm to stretch	5/10	10/10

PSFS, Patient Specific Functional Scale.
